# Genotype Load Modulates Amyloid Burden and Anxiety-Like Patterns in Male 3xTg-AD Survivors despite Similar Neuro-Immunoendocrine, Synaptic and Cognitive Impairments

**DOI:** 10.3390/biomedicines9070715

**Published:** 2021-06-23

**Authors:** Aida Muntsant, Lydia Giménez-Llort

**Affiliations:** 1 Institut de Neurociències, Universitat Autònoma de Barcelona, 08193 Barcelona, Spain; aida.muntsant@uab.cat; 2 Department of Psychiatry and Forensic Medicine, School of Medicine, Universitat Autònoma de Barcelona, 08193 Barcelona, Spain

**Keywords:** Alzheimer’s disease, genetic load, survival, end-of-life, frailty, heterogeneity, BPSD, NPS, neuro-immunoendocrine crosstalk

## Abstract

The wide heterogeneity and complexity of Alzheimer’s disease (AD) patients’ clinical profiles and increased mortality highlight the relevance of personalized-based interventions and the need for end-of-life/survival predictors. At the translational level, studying genetic and age interactions in a context of different levels of expression of AD-genetic-load can help to understand this heterogeneity better. In the present report, a singular cohort of long-lived (19-month-old survivors) heterozygous and homozygous male 3xTg-AD mice were studied to determine whether their AD-genotype load can modulate the brain and peripheral pathological burden, behavioral phenotypes, and neuro-immunoendocrine status, compared to age-matched non-transgenic controls. The results indicated increased amyloid precursor protein (APP) levels in a genetic-load-dependent manner but convergent synaptophysin and choline acetyltransferase brain levels. Cognitive impairment and HPA-axis hyperactivation were salient traits in both 3xTg-AD survivor groups. In contrast, genetic load elicited different anxiety-like profiles, with hypoactive homozygous, while heterozygous resembled controls in some traits and risk assessment. Complex neuro-immunoendocrine crosstalk was also observed. Bodyweight loss and splenic, renal, and hepatic histopathological injury scores provided evidence of the systemic features of AD, despite similar peripheral organs’ oxidative stress. The present study provides an interesting translational scenario to study further genetic-load and age-dependent vulnerability/compensatory mechanisms in Alzheimer’s disease.

## 1. Introduction

Alzheimer’s disease (AD) is the leading cause of dementia, one of the principal causes of disability in late adulthood. It is a multi-factorial disorder caused by the interaction of biological, environmental factors, where age-related changes play a determinant role [1]. At the neuropathological level, Alzheimer’s disease is mainly defined by an extracellular accumulation of amyloid-β (Aβ) plaques and reactive gliosis and cellular tau-containing neurofibrillary tangles (NFTs) accompanied by synaptic dysfunction and cholinergic-dependent progressive memory decline [2]. Whereas cognitive dysfunction defines the diagnosis of the core clinical symptoms, neuropsychiatric symptoms (NPS) [3], also called Behavioral and Psychological Symptoms of Dementia (BPSD), are observed in 90% of patients. The broad array of NPS include agitation, anxiety, verbal, or physical aggression, sundowning behavior, wandering, depression, challenging and disruptive behaviors, hallucinations, among other alterations [4]. These NPS are highly associated with the burden of disease, lower quality of life and caregiver burnout [5], and earlier institutionalization [6].

Neurodegenerative disorders such as dementia are associated with increased mortality compared to aged control populations [7,8,9]. Although sex-specific clinicopathological mechanism is not well understood and are largely unexplored [10], males presented deranged neuro-immuno-endocrine system despite their less harmful neuropathological status [11,12]. Moreover, the vast heterogeneity and complexity of patients’ clinical profiles and temporal progression of the disease highlight the relevance of personalized-based interventions [13]. However, despite there is more than one potential therapeutic target for this disease, currently approved interventions are just a few; they target loss of cholinergic function and excitotoxicity but exert modest benefits restricted to symptomatology [14]. Disease-modifying treatments are still under intensive research and development [15]. Better understanding the implication of genetic and phenotypic factors may also provide novel mechanisms for clustering AD patients [16,17] and would be determinant also when translated to experimental models [18].

Thanks to the shorter life span of most non-human animals, translational research can provide a fleet-footed scenario for studying genetic and age interactions in the context of different levels of AD-genetic expression. Also, this short temporal frame is very appreciated for long-term monitoring and study of factors potentially involved in disease modulation from morphological, structural, functional, and behavioral levels. Among the animal models of AD, we have proposed long-term survivors of the widely used 3xTg-AD mice as a model for heterogeneity in end-of-life dementia [19]. This model, based on the familial AD mutations PS1/M146V and APPSwe, also harboring the tauP301L human transgene, progressively develops temporal- and regional-specific development of amyloid-β plaques and tau-containing neurofibrillary tangles observed in the human brain of AD patients [20,21]. It also mimics other hallmarks of the disease such as synaptic dysfunction and decreased long-term potentiation [21,22], neuroinflammation, reactive gliosis [23,24], brain oxidative stress [25,26] as well as changes in neurotransmitter systems [27] and impairment in neuro-immunoendocrine status [28]. Cognitive deficits [29,30] and a wide spectrum of neuropsychiatric (NPS)-like disturbances have also been described [18,31,32]. Whether they are sensitive to genetic-load and in which way remains to be determined.

Since the establishment of 3xTg-AD and NTg mouse colonies in our laboratory we have repeatedly observed that the animals overcoming 15 months of age show milder impairment of cognitive and NPS-like behaviors than expected for their neuropathological status. Females usually presented greater survivors’ rates than males, for this reason we have previously described behavioral and functional phenotype of long-term survivors, 18-month-old female 3xTg-AD mice [19]. The present work studied a singular cohort of long-lived (19-month-old survivors) heterozygous and homozygous male 3xTg-AD with an extraordinary survival rate. In this particular scenario, we explored how the AD-genetic load interferes with the normal aging scenario and its implication in the pathological burden and neuro-immunoendocrine status involving not only the HPA axis but also peripheral organs. Finally, how the genetic load translates into these survivors’ cognitive and NPS behavioral phenotype was also explored. These results highlight that amyloid precursor protein (APP) levels increased in a genetic load-dependent manner, but similar synaptophysin and choline acetyltransferase brain levels. Cognitive impairment was invariable as the distinct trait of Alzheimer’s disease; however, anxiety-like behavior seemed more related to the genetic-load. Convergence of physical status and sensorimotor profiles were more related to normal aging processes. On the other hand, complex neuro-immunoendocrine crosstalk was observed with peripheral histopathology, but no correlations with frailty index nor oxidative stress parameters were found. These results suggest the existence of vulnerability/compensatory mechanisms in transgenic mice.

## 2. Materials and Methods

### 2.1. Animals

Homozygous triple-transgenic 3xTg-AD mice harboring human PS1/M146V, APPSwe, and tauP301L transgenes were genetically engineered at the University of California Irvine, as previously described [21]. Briefly, two independent transgenes (encoding human APPSwe and human tauP301L, both under control of the mouse Thy1.2 regulatory element) were co-injected into single-cell embryos harvested from homozygous mutant PS1M146V knock-in (PS1KI) mice. The PS1 knock-in mice were originally generated after embryonic transfer into pure C57BL/6.

A cohort of seventeen mice from the Spanish colonies of 3xTg-AD (*n* = 9, homozygous, *n* = 5 heterozygous, *n* = 4) and C57BL/6 (*n* = 8) wild-type mice (from now, referred as non-transgenic mice, NTg) from litters of a breeding program established after embryonic transfer to C57BL/6 strain background were used in this study. All animals were housed and maintained (Makrolon, 35 × 35 × 25 cm^3^) under standard laboratory conditions (12 h light/dark, cycle starting at 8:00 a.m., food and water ad libitum, 22 ± 2 °C, 50–60% humidity) at Universitat Autònoma de Barcelona. Behavioral tests were performed from 9:00 h to 13:00 h. Behavioral assessments, biochemical and neuropathological analyses were performed blind to the experiment in a counterbalanced manner. 

All procedures followed Spanish legislation on ‘Protection of Animals Used for Experimental and Other Scientific Purposes’ and the EU Council directive (2010/63/EU) on this subject. The protocol CEEAH 3588/DMAH 9452 was approved the 8 March 2019 by Departament de Medi Ambient i Habitatge, Generalitat de Catalunya. The study complies with the ARRIVE guidelines developed by the NC3Rs and aims to reduce the number of animals used [33].

### 2.2. Experimental Design 

At 18 months of age, mice’s physical and mental health status started to be characterized and concluded at 19 months [18.70 ± 0.17 (17.5–19.31)]. After that, samples for physiological, biochemical, and pathological analysis were collected. Survival was continuously monitored.

### 2.3. Behavioral Assessment

Comprehensive screening of several physical, emotional, and cognitive functions was successively performed using a battery of tests based on three main behavioral dimensions that can be described as follows:

#### 2.3.1. Physical Status, Reflexes, and Sensorimotor Functions 

Visual reflex and posterior leg extension reflex were measured three times by holding the animal by the tail and slowly lowering it to a black surface. Motor coordination (distance covered) and equilibrium (latency to fall off) were assessed in a horizontal wood (1.3 cm wide) and a metal (1 cm diameter) rod on two consecutive 20 s trials each. Motor coordination (mean distance covered) and muscle strength (latency to fall off in the two 5 s trials) and motor strength (latency to fall off the 60 s trial) were measured in the wire hang test consisting of allowing the animal to cling from the middle of a horizontal wire (diameter: 2 mm, length: 40 cm, divided into eight 5 cm segments) with the forepaws for two trials of 5 s and a third 60 s trial. All the apparatuses were suspended 40 cm above a padded table.

#### 2.3.2. Neuropsychiatric-Like Behaviors

Changes in emotionality increased neophobia and other signs of anxiety-like responses, all of them BPSD-like behaviors modeled in 3xTg-AD mice [18], were measured in classical unconditioned tests. The tests evaluate locomotion/exploration, anxiety-like behaviors, and emotionality under different anxiogenic conditions.

##### Corner Test (CT) and Open Field Test (OF)

Neophobia was assessed in the corner test for 30 s, placing the animal in the center of a clean standard home cage filled with wood save bedding. The number of corners visited, the latency of first rearing and the number of rearings were recorded. [18]. Immediately after the CT, mice were placed in the center of an open field (metalwork, white box, 42 × 38 × 15 cm) and observed for 5 min [34]. The ethogram, described by the temporal profile of the following sequence of behavioral events, was recorded: duration of freezing behavior, latency to leave the central square, and that of entering the peripheral ring and latency and total duration of self-grooming behavior. Horizontal (crossings of 10 × 10 cm squares) and vertical (rearings with wall support) locomotor activities were also measured. During the tests, defecation boli and urination were also recorded. The repeated test, 24 h later, was used to evaluate the long-term memory of these experiences [35]. Distance and time in the center/periphery were evaluated by video-tracking analysis (ANY-Maze, version 5.14, Stoelting Europe, Dublin, Ireland).

##### Dark–Light Box Test (DLB)

Anxiety and risk assessment were measured for 5 min after introducing the animal into the dark compartment of the DLB (Panlab S.L., Barcelona, Spain). The apparatus consisted of two compartments (black, 27 cm × 18 cm × 27 cm^3^, white, 27 cm × 27 cm × 27 cm^3^ illuminated by a red 20 W bulb) connected by an opening (7 cm × 7 cm^2^). The experimental room was kept in darkness. Latency to enter the lit compartment, the number of entries, total time spent, distance covered, and the number of rearings and groomings in this compartment were noted. Risk assessment was measured as the latency and number of stretch attendances toward the lit area.

##### Marble Test (MB)

The animal was placed in a standard home cage containing nine glass marbles (1 × 1 × 1 cm^3^) evenly spaced, making a square (three rows of three marbles per row only in the left area of the cage) on a 5 cm thick layer of sawdust. The mice were left in the cage with marbles for a 30 min period, after which the test was terminated by removing the mice and counting the number of marbles: intact (untouched), rotated (90 or 180°), half-buried (at least ½ buried by sawdust), and buried (completely hidden).

#### 2.3.3. Cognitive Function

##### T-Maze Test (TM)

Two different paradigms were carried out in a T-shaped maze (woodwork; two short arms of 30 × 10 cm^2^; one long arm of 50 × 10 cm^2^). Coping with stress strategies, risk assessment, and working memory were assessed in the spontaneous alternation task [36]. The animal was placed inside the maze’s long arm with its head facing the end wall, and it was allowed to explore the maze for a maximum of 5 min. The ethogram (latencies to different goals) in this task was recorded as follows: to move and turn (freezing behavior), to reach the intersection, to cross (4 paws criteria) the intersection of the three arms, and the total time invested in exploring the three arms of the maze (test completion criteria). 

On the day after, the second working memory paradigm consisted of one forced-choice followed, 60 s later, by one free choice (recall trial). Here, goals were defined as: to move and turn, to reach the intersection, the time elapsed until the animal crossed (4 paws criteria) the intersection of the three arms, and the time elapsed until the mice completed 20 s in the forced arm (time to reach the criteria). The animals that completed the forced trial in less than the cut-off time (10 min) were allowed to explore the maze in the free choice trial where both arms were accessible for 5 min. The arm chosen by the mice and the time spent to reach the correct arm during the free choice were recorded (exploration criteria). 

In both paradigms, the choice of the already visited arm in the previous trial was considered an error, and the total number was calculated. Finally, defecation boli and urination were also recorded.

##### Morris-Water-Maze (MWM)

Three learning and memory paradigms were administered during 5 consecutive days. First, mice were trained to locate a hidden platform (7-cm diameter, 1 cm on/below the water surface) in a circular pool for mice (120 cm in diameter and 60 cm deep, 25 °C opaque water). Mice that failed to find the platform within 60 s were placed on it for 10 s, the same period was allowed for the successful animals. 

Cue learning with a visible platform: On the first day, the animals were tested for the cued learning of a visual platform consisting of four trials in 1 day. In each trial, the mouse was gently released (facing the wall) from one randomly selected starting point (W-S-E-N) and allowed to swim until it escaped onto the platform, elevated 1 cm above the water level in the NE position and indicated by a visible striped flag (5.3 × 8.3 × 15 cm^3^). Extra maze cues were absent in the black walls of the room. 

Place learning with a hidden platform: On the following day, the place learning task consisted of four trial sessions per day for 4-days with trials spaced 30 min apart. The mouse was gently released (facing the wall) from one randomly selected starting point (N-E-W-S; E-N-S-W) and allowed to swim until it escaped onto the hidden platform, which was now located in the middle of the SW quadrant (reversal). Different geometric figures hung on each wall of the room were used as external visual clues. Variables of time (escape latency), distance covered, and swimming speed were analyzed in all the tasks’ trials. The escape latency was readily measured with a stopwatch by an observer unaware of the animal’s genotype and confirmed during the subsequent video-tracking analysis (ANY-Maze v. 5.14, Stoelting, Dublin, Ireland). 

Two hours and 30 min after the last place task, the platform was removed, and the animal was allowed to swim for a 60 s probe trial. Quadrant preference and entries into the previous platform location were video-tracked and analyzed.

### 2.4. Body Weight, Mouse Clinical Frailty Index Assessment, and Survival 

Bodyweight and frailty were assessed using an adaptation of the MCFI [37], including 30 clinically-like assessed non-invasive items. For 29 of these items, mice were given a score 0 if not presented, 0.5 if there was a mild deficit, and 1 for the severe deficit. Weight was scored based on the number of standard deviations from a reference mean. The clinical evaluation included the integument, the physical/musculoskeletal system, the vestibulocochlear/auditory systems, the ocular and nasal systems, the digestive system, the urogenital system, the respiratory system, signs of discomfort, and body weight. Survival was recorded continuously with a daily cadence.

### 2.5. HPA Axis Endocrine Status

Four days after the behavioral assessment, blood samples were collected during the euthanasia. Serum was obtained by centrifugation and stored at −80 °C. Corticosterone content (ng/mL) was analyzed using a commercial kit (Corticosterone EIA Immunodiagnostic Systems Ltd., Boldon, UK) and read at 450 nm of absorbance with Varioskan LUX ESW 1.00.38. (Thermo Fisher Scientific, Waltham, MA, USA).

### 2.6. Neuropathology and Synaptic Function

Brain and peripheral organs were dissected for further biochemical and/or pathological analysis. The right prefrontal cortex, entorhinal cortex, and hippocampus were dissected out, weighed, snap-frozen separately, and stored at –80 °C until processing for preparation of protein extract for biochemistry analysis. Frozen samples were lysed in cold lysis buffer containing protease and phosphatase inhibitors (Sigma-Aldrich, Saint Louis, MO, USA). Protein content was quantified with the BCA Protein Assay Kit (Thermo Fisher Scientific, Waltham, MA, USA), resolved on SDS-polyacrylamide gel electrophoresis, and detected by Western blotting using the following antibodies: 6E10 (1:500, Biolegend, San Diego, CA, USA); synaptophysin (1:2000, Dako, Glostrup, Denmark); ChAT (1:500, Thermo Fisher Scientific, Waltham, MA, USA), β-actin (1:10,000; Sigma-Aldrich, Saint Louis, MO, USA). Bands were detected with an enhanced chemiluminescent reagent in a ChemiDoc MP System (Bio-Rad Laboratories, Inc., Hercules, CA, USA) and quantified in a linear range using the ImageLab 5.2.1 software (Bio-Rad Laboratories, Inc., Hercules, CA, USA).

### 2.7. Peripheral Organs Pathological Status

#### 2.7.1. Splenic, Renal and Hepatic Histopathological Evaluation 

Spleen, kidneys, and liver were dissected and port-fixed by immersion 24 h in 10% formalin (Sigma-Aldrich, Saint Louis, MO, USA). The size (weight in mg) and organ indexes (relative size, % vs. bodyweight) of the spleen were also recorded as an indirect measure of the physiological status of the peripheral immune organs. All samples were washed twice with 4 °C phosphate buffered saline for about 20 min to stop fixation and rinse the fixative, immersed in ethanol 70% and kept at 4 °C until paraffin embedding. Histological processing for paraffin embedding was performed by means of an automatic processing machine (Leica TP1020, Leica Biosystems, Nussloch, Germany): EtOH 70% 30 min; EtOH 80% 20 min; EtOH 96% 2 × 20 min; EtOH 100% 2 × 30 min + 1 × 40 min; EtOH 100%-Xylene 30 min; Xylene 2 × 40 min; paraffin 2 × 1 h. The paraffin blocks were confectioned in a paraffin embedding station (Leica EG1150H, Leica Biosystems, Nussloch, Germany) and were cooled in a cold plate (Leica EG1150C, Leica Biosystems, Nussloch, Germany). Histological preparations of spleen, kidney, and liver were stained with hematoxylin-eosin, and a pathological evaluation was performed blindly by an expert pathologist following a criterion based on the intensity and the distribution of the lesion. The degree of tissue damage was calculated following an injury score grading system: 0—no damage, 1: mild, 2: moderate, 3: severe, 4: very severe. As the same basic lesion of amyloid characteristics was observed in the different organs, a systemic character of the damage was confirmed, and a total injury score with the different organs damage was used to evaluate the total systemic injury.

#### 2.7.2. Oxidative Stress of Spleen, Kidneys, Liver, and Heart

The antioxidant capacity was studied from the evaluation of the levels of total glutathione (GSH), as well as the enzymatic activity of glutathione peroxidase (GPx) and reductase (GR) from the homogenization of different organs (liver, kidneys, spleen, and heart).

Glutathione concentrations: Total glutathione, the main non-enzymatic reducing agent of the organism, was assayed by the enzymatic recycling method previously described [38] by monitoring the change in absorbance at 412 nm adapted to 96-well plates with slight modifications [39].

Glutathione reductase activity: The activity of the enzyme glutathione was assessed following the method described by Massey and Williams [40] with slight modifications. The total activity was determined following the oxidation of NADPH spectrophotometrically at 340 nm for 300 s. The results were expressed as milliunits (mU) of enzymatic activity per mg of organ protein. 

Glutathione peroxidase activity: The glutathione peroxidase activity was measured using the modified technique previously described [41,42] with slight modifications. The reaction was followed spectrophotometrically by the decrease of the absorbance at 340 nm for 300 s. The results were expressed as mU of enzymatic activity per mg of organ protein.

### 2.8. Statistics

Results are expressed as mean ± SEM. SPSS 15.0 (SPSS Inc., Chicago, IL, USA) and GraphPad Prism 8.0 (GraphPad Software Inc., San Diego, CA, USA) software were used. Differences between two different genotypes were evaluated with a two-tailed unpaired Student’s *t*-test (U Mann Whitney, for quantitative discontinuous). One-way analysis of variance (ANOVA) for comparisons between all the groups of mice, including NTg, 3xTg-AD homozygous, and 3xTg-AD heterozygous, followed by Bonferroni’s post hoc test. In the temporal courses, RMA, Repeated measures ANOVA, was used for within-subject analysis. Differences in life spans were studied through the Kaplan-Meier test. In all the tests, statistical significance was considered at *p* < 0.05.

## 3. Results

**Figure 1:**
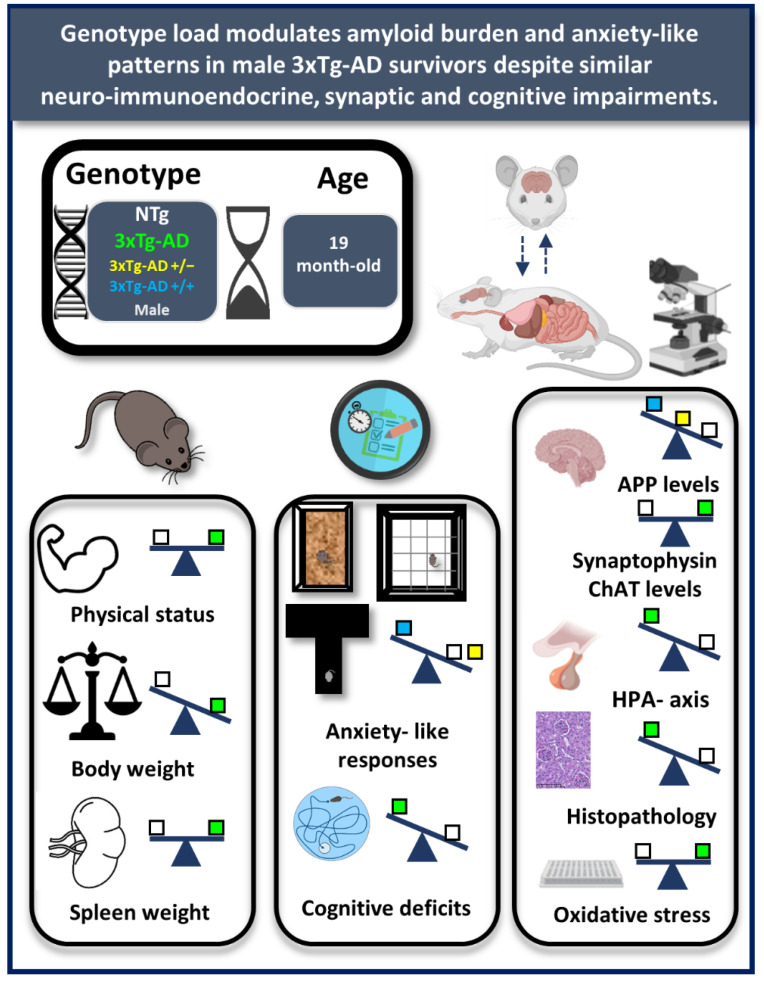
Graphical abstract. Genotype load modulates amyloid burden and anxiety-like patterns in male 3xTg-AD survivors despite similar neuro-immunoendocrine and cognitive impairments. Experimental design and main findings. White square: NTg mice, yellow square: heterozygous 3xTg-AD mice, blue square: homozygous 3xTg-AD mice; Green square: 3xTg-AD mice (both 3xTg-AD genotypes, since no genotype-load differences were found).

**Figure 2:**
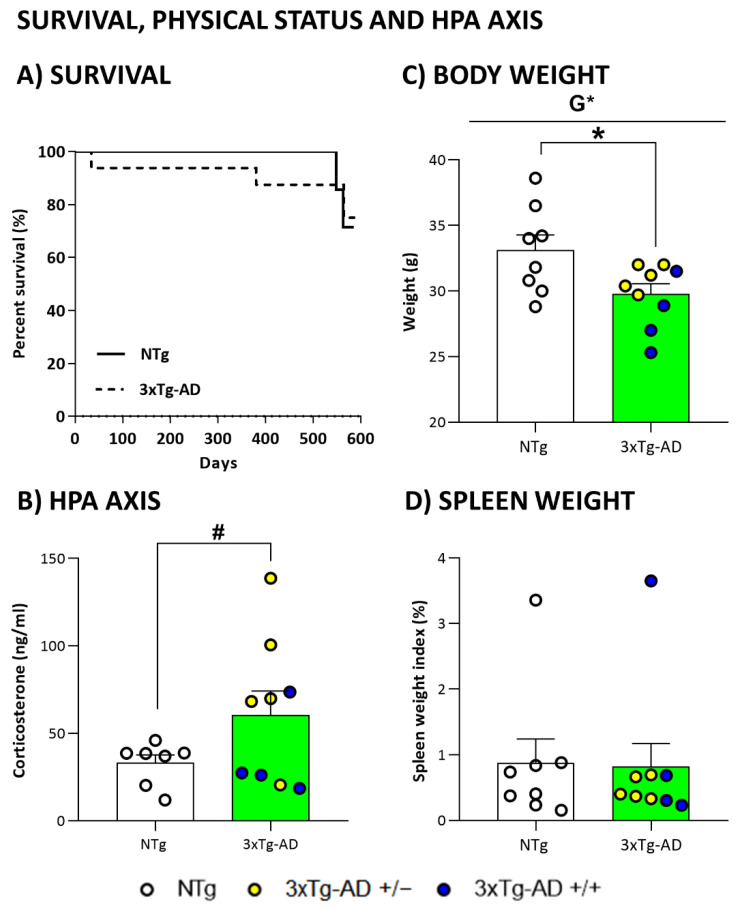
Survival, HPA axis endocrine status and physical health. (**A**) Survival; (**B**) Corticosterone levels; (**C**) Body weight and (**D**) spleen weight in 19-month-old mice. Results are expressed as the mean ± SEM. Male NTg, *n* = 8 (White circles for individual values; white bar, mean value); male 3xTg-AD *n* = 9 (Yellow circles, individual values of heterozygous 3xTg-AD +/− mice, *n* = 5; Blue circles, individual values for homozygous 3xTg-AD +/+ mice, *n* = 4; Green bar, mean value of both 3xTg-AD genotypes, since no genotype-load differences were found). Statistics: two-tailed unpaired Student’s *t*-test (above the line) for Genotype differences (G): * *p* < 0.05; one-way analysis of variance (ANOVA) for comparisons between all the groups of mice followed by Bonferroni’s post-hoc test. * *p* < 0.05 vs. homozygous 3xTg-AD-group; # *p* < 0.05 vs. heterozygous 3xTg-AD-group.

**Figure 3:**
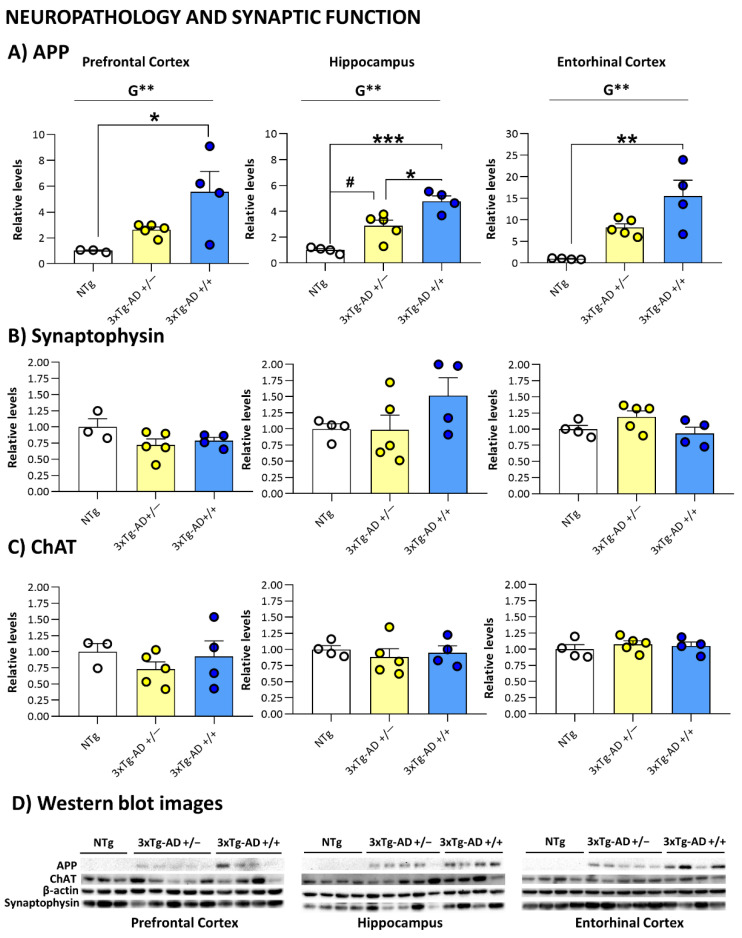
Neuropathology and Synaptic Function: Brain biochemical analysis of APP (**A**), synaptophysin (**B**) and ChAT (**C**) in the prefrontal cortex, hippocampus, and entorhinal cortex. (**D**) Western blot images. Results are expressed as the relative levels as fold change ± SEM. Male NTg, *n* = 3–4 (White circles for individual values; white bar, mean value); male 3xTg-AD *n* = 9 (Yellow circles, individual values of heterozygous 3xTg-AD +/− mice, *n* = 5; blue circles, individual values for homozygous 3xTg-AD +/+ mice, *n* = 4; yellow and blue bars, mean value for heterozygous and homozygous 3xTg-AD genotypes, respectively, since genotype-load differences were found). Statistics: two-tailed unpaired Student’s *t*-test (above the line) for Genotype differences (G): ** *p* < 0.01; one-way analysis of variance (ANOVA) for comparisons between all the groups of mice followed by Bonferroni’s post hoc test. * *p* < 0.05, ** *p* < 0.01, *** *p* < 0.001 vs. homozygous 3xTg-AD-group; # *p* < 0.05 vs. heterozygous 3xTg-AD-group.

**Figure 4:**
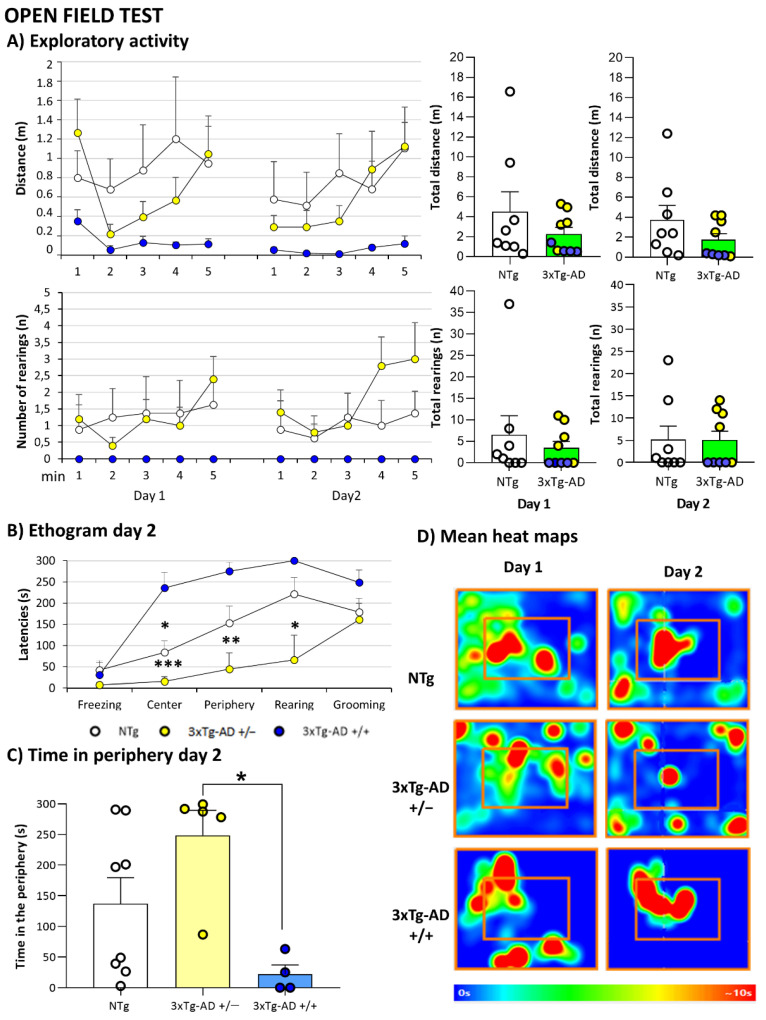
Mental Health: Neuropsychiatric-like phenotype in 2-day Open-field test. (**A**) Exploratory activity; (**B**) Ethogram day 2; (**C**) Time in periphery day 2 and (**D**) Heat maps representation of how much time animals spends in different parts of the apparatus during a test, with blue as the shortest time and red as the longest. Results are expressed as the mean ± SEM. Male NTg, *n* = 8 (White circles for individual values; white bar, mean value); male 3xTg-AD *n* = 9 (Yellow circles, individual values of heterozygous 3xTg-AD +/− mice, *n* = 5; blue circles, individual values for homozygous 3xTg-AD +/+ mice, *n* = 4; green bar, mean value of both 3xTg-AD genotypes, since no genotype-load differences were found; yellow and blue bars, mean value for heterozygous and homozygous 3xTg-AD genotypes, respectively, since genotype-load differences were found). Statistics: one-way analysis of variance (ANOVA) for comparisons between all the groups of mice followed by Bonferroni’s post hoc test. * *p* < 0.05, ** *p* < 0.01, *** *p* < 0.001 vs. homozygous 3xTg-AD-group.

**Figure 5:**
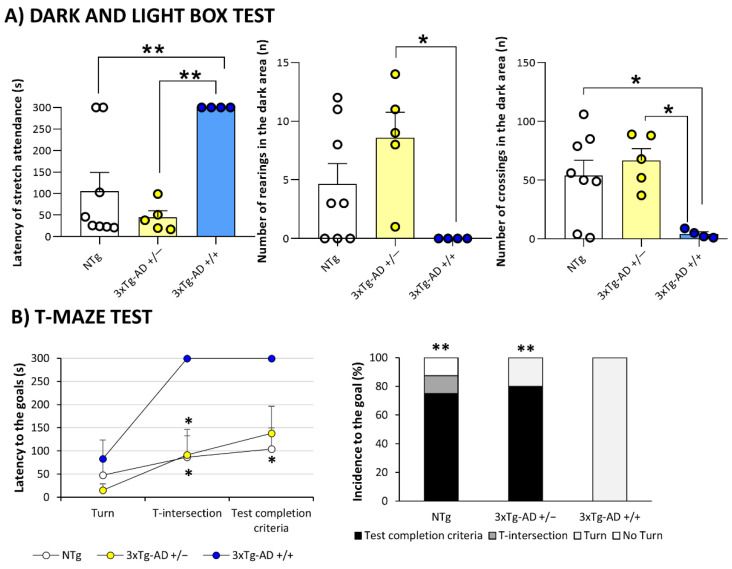
Mental Health: Neuropsychiatric-like phenotype and cognitive impairment in Dark and light box test (**A**) and T-Maze test (**B**). Results are expressed as the mean ± SEM. Male NTg, *n* = 8 (White circles for individual values; white bar, mean value); male 3xTg-AD *n* = 9 (Yellow circles, individual values of heterozygous 3xTg-AD +/− mice, *n* = 5; blue circles, individual values for homozygous 3xTg-AD +/+ mice, *n* = 4; yellow and blue bars, mean value for heterozygous and homozygous 3xTg-AD genotypes, respectively, since genotype-load differences were found). Statistics: one-way analysis of variance (ANOVA) for comparisons between all the groups of mice followed by Bonferroni’s post hoc test. * *p* < 0.05, ** *p* < 0.01, vs. homozygous 3xTg-AD-group.

**Figure 6:**
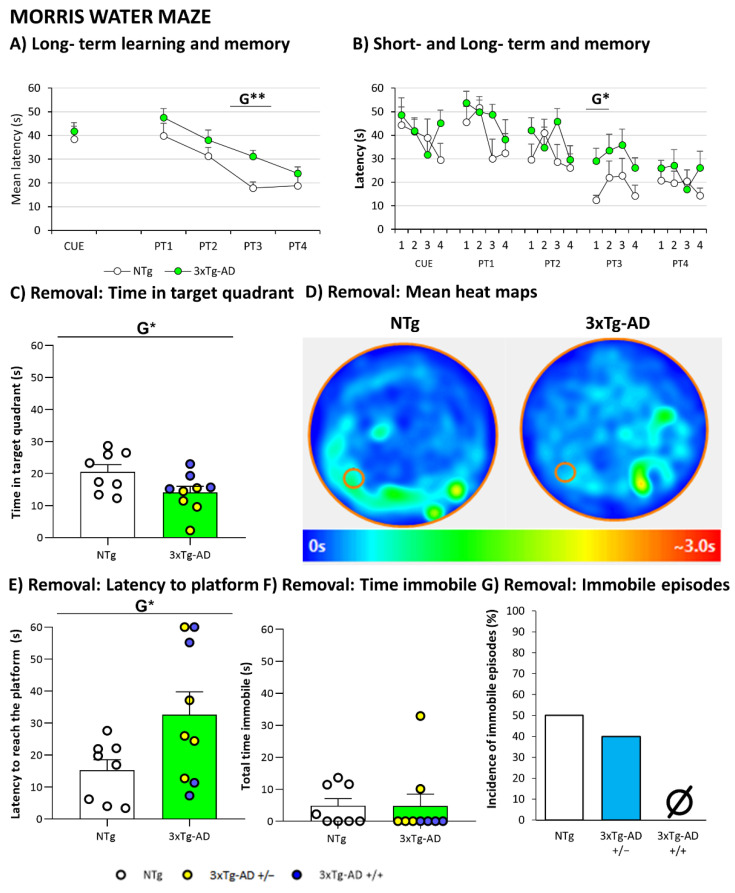
Mental Health: Cognitive impairment in Morris Water Maze (**A**) Long-term learning and memory; (**B**) Short- and long-term memory; (**C**) Short-term memory in Removal: Time in target quadrant; (**D**) Heat maps representation in removal. (**E**) Short-term memory in Removal: Latency to reach the platform; (**F**) Time immobile in removal; (**G**) Immobile episodes in removal. Results are expressed as the mean ± SEM. Male NTg, *n* = 8 (White circles for individual values; white bar, mean value); male 3xTg-AD *n* = 9 (Yellow circles, individual values of heterozygous 3xTg-AD +/− mice, *n* = 5; blue circles, individual values for homozygous 3xTg-AD +/+ mice, *n* = 4; green bar, mean value of both 3xTg-AD genotypes, since no genotype-load differences were found; yellow and blue bars, mean value for heterozygous and homozygous 3xTg-AD genotypes, respectively, since genotype-load differences were found). Heat maps representation of how much time animals spends in different parts of the apparatus during a test, with blue as the shortest time and red as the longest. Statistics: two-tailed unpaired Student’s *t*-test (above line) for Genotype differences (**G**): * *p* < 0.05, ** *p* < 0.01.

**Figure 7:**
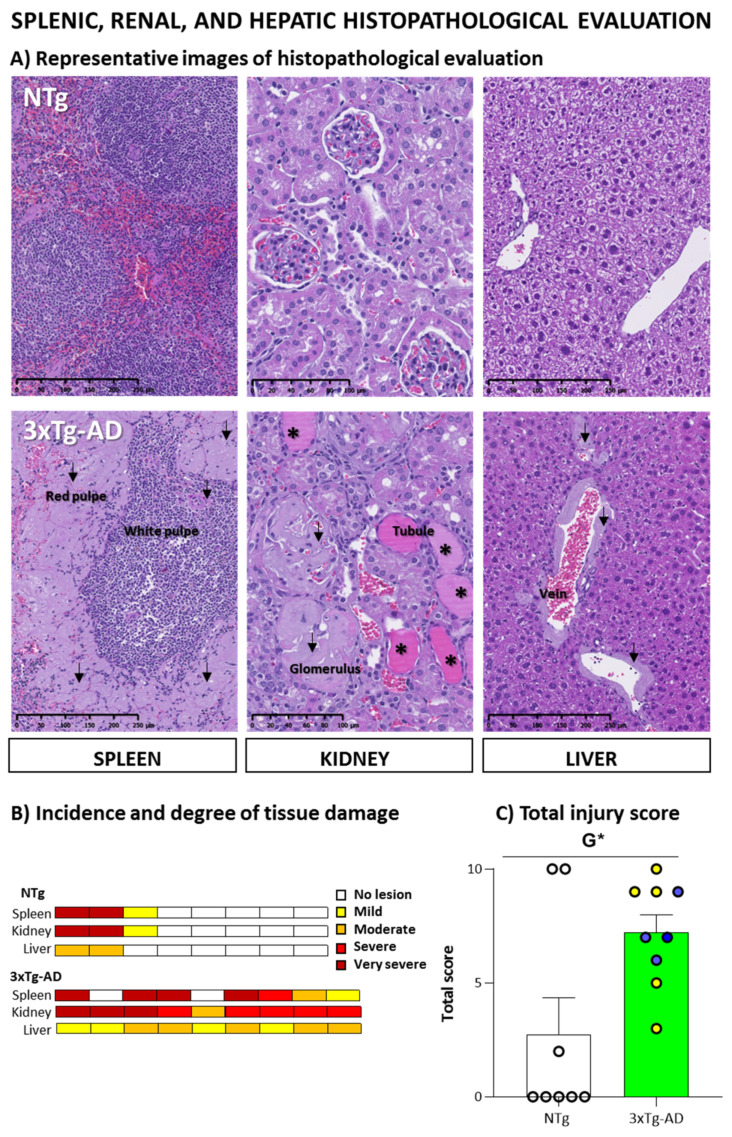
Pathological Status of Peripheral Organs in 19-month-old male 3xTg-AD mice and NTg counterparts: (**A**) Representative images of histopathological evaluation of peripheral organs (kidney, spleen, and liver), Black arrow: amyloidosis damage, *: proteinuria; (**B**) Incidence and degree of tissue damage; (**C**) General systemic total score. Results are expressed as the mean ± SEM. Male NTg, *n* = 8 (White circles for individual values; white bar, mean value); male 3xTg-AD *n* = 9 (Yellow circles, individual values of heterozygous 3xTg-AD +/− mice, *n* = 5; Blue circles, individual values for homozygous 3xTg-AD +/+ mice, *n* = 4; Green bar, mean value of both 3xTg-AD genotypes, since no genotype-load differences were found). Statistics: two-tailed unpaired Student’s *t*-test (above line) for Genotype differences (G): * *p* < 0.05.

**Figure 8:**
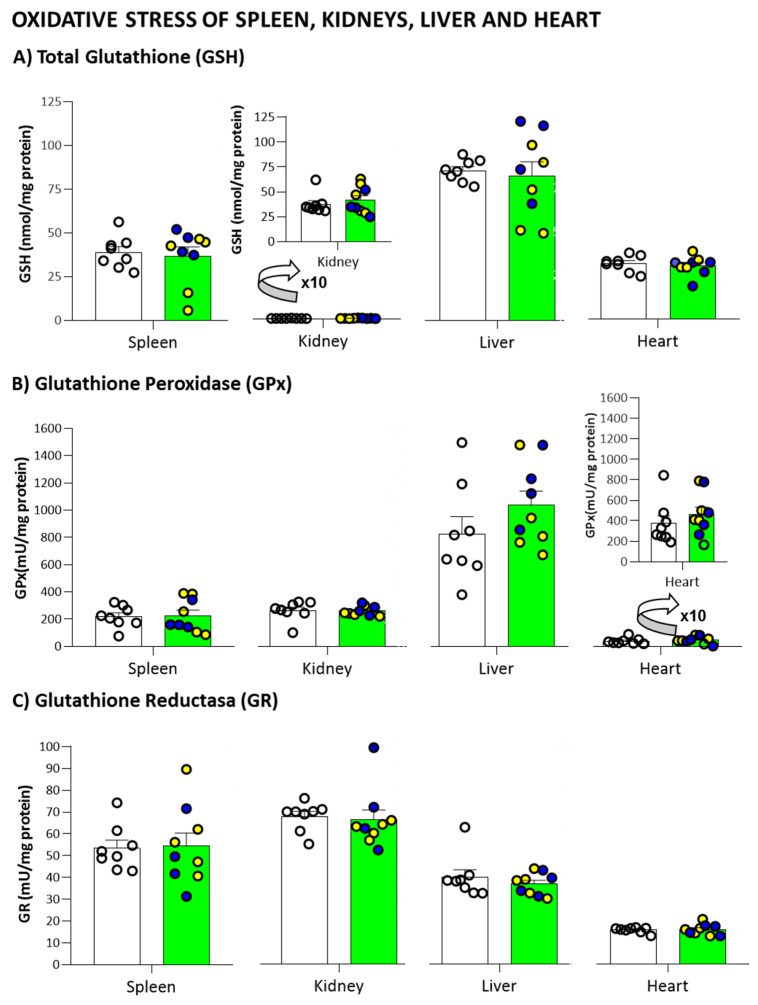
Pathological Status of Peripheral Organs: Oxidative stress parameters in peripheral organs: (**A**) Total Glutathione (GSH); (**B**) Glutathione Peroxidase (GPx); (**C**) Glutathione Reductase (GR) in 19-month-old male 3xTg-AD mice and NTg counterparts. Results are expressed as the mean ± SEM. Male NTg, *n* = 8 (White circles for individual values; white bar, mean value); male 3xTg-AD *n* = 9 (Yellow circles, individual values of heterozygous 3xTg-AD +/− mice, *n* = 5; Blue circles, individual values for homozygous 3xTg-AD +/+ mice, *n* = 4; Green bar, mean value of both 3xTg-AD genotypes, since no genotype-load differences were found). Statistics: n.s., no significative, *p* > 0.05.

As summarized in the graphical abstract (Figure 1) and depicted in Figure 2, Figure 3, Figure 4, Figure 5, Figure 6, Figure 7 and Figure 8, the main findings show that genotype-load modulated amyloid burden and anxiety-like patterns in male 3xTg-AD survivors despite similar neuro-immunoendocrine and cognitive impairments.

### 3.1. Survival

Survival curves of an initial sample of 28 male, twelve NTg, and sixteen 3xTg-AD mice are illustrated in Figure 2A. Although 3xTg-AD mice showed a younger mortality pattern than NTg mice, Log-rank analyses showed no differences when starting the experiment (71.4% in NTg vs. 87.5% in 3xTg-AD mice). During the experiment, one 3xTg-AD died. At the end of the experiment, the survival rates were 71.4% in NTg vs. 75% in 3xTg-AD mice.

### 3.2. HPA Axis Endocrine Status

Although no significant differences were observed between genotypes when we measured the corticosterone values (Figure 2B), heterozygous mice showed a higher level in comparison NTg mice (*p* = 0.045, post hoc test).

### 3.3. Physical Status, Reflexes, and Sensorimotor Functions

As detailed in Table 1, frailty index, reflexes, and sensorimotor functions of 19-month-old 3xTg-AD mice did not differ from those observed in NTg mice with normal aging (genotype effects, Student *t*-test, *p* > 0.05 or even equal values). Independently of genotype, animals did not cover almost any distance, and latencies were very short. In both groups, the measures of resistance and coordination revealed high individual variability. Genotype differences were found in body weight (Figure 2C), increased in 3xTg-AD mice (*p* = 0.037, Student’s *t*-test). However, this difference was only observed between homozygous and NTg mice (*p* = 0.032, post hoc test). No differences in spleen weight were observed (Figure 2D).

### 3.4. Neuropathology and Synaptic Function

The analysis of immunoblotting from the prefrontal cortex, hippocampus, and entorhinal cortex protein extracts incubated with 6E10 antibody showed a genetic-load-dependent increase of APP (Figure 3A); (*p* < 0.008, Student’s *t*-test). This gradient was mainly observed in the hippocampus, where significant differences were also observed between homozygous and heterozygous groups (*p* < 0.016, Bonferroni’s post hoc test, homozygous vs. NTg and heterozygous mice). However, no differences (*p* > 0.05, Student’s *t*-test) were found when we evaluate levels of synaptophysin (Figure 3B) a synaptogenesis and neuroplasticity marker nor choline acetyltransferase (Figure 3C), the enzyme responsible for the acetylcholine synthesis.

### 3.5. Neuropsychiatric Symptoms (NPS)-Like Phenotype and Cognitive Impairment under Different Anxiogenic Conditions

In the corner test, genotype differences were observed only when horizontal activity was measured in the first corner test (*p* = 0.002, Student’s *t*-test) and disappeared when we repeated the test 24 h later. Although the activity between tests was decreased in all groups, statistical differences were observed in the number of corners in NTg mice (*p* = 0.009, paired *t*-test) and the number of rearings in 3xTg-AD mice (*p* = 0.038, paired *t*-test). Surprisingly, results in the open field test evidenced striking similarities between 18-month-old 3xTg-AD and NTg mice. Thus, all the analyzed behavioral variables (see Table 2 and Figure 4), including those related to the time course of elicitation of behavioral events and emotionality. Furthermore, as observed in the physical and sensorimotor aspects, both groups of mice showed high individual variability, resulting in high statistical variance. However, when we repeated the test 24 h later, significant differences in open field ethogram were observed between homozygous and heterozygous mice (*p* < 0.015, post hoc test). Lower latencies were observed in the exit of the center, entrance to the periphery, and vertical activity in heterozygous mice (Figure 4B). Moreover, heterozygous mice spent more time in the periphery than homozygous mice (*p* = 0.013, post hoc test, Figure 4C,D).

In the Dark light box, no genotype differences were observed between 3xTg-AD and NTg (*p* > 0.05, Student’s *t*-test). However, when we evaluated homozygous and heterozygous independently, significant differences were observed (Figure 5A). Albeit did not reach statistical significance, none of the homozygous mice entered into the lit area. Besides, during the 300 s of the test, homozygous did not perform any stretch attendance than NTg and heterozygous mice (*p* < 0.009, post hoc test). Moreover, they did not perform any rearing in the dark area during the test (*p* = 0.033, post hoc test vs. heterozygous mice). The number of crossing was also reduced (*p* < 0.043, post hoc test vs. heterozygous and NTg mice).

In the Marble test, no genotype differences were observed between 3xTg-AD and NTg in the Marble test (*p* > 0.05, Student’s *t*-test), as indicated in Table 2.

In the T-maze, no genotype differences were observed between 3xTg-AD and NTg in both T-maze test. However, when we evaluated the two 3xTg-AD subgroups (Figure 5B), we observed that when we studied the coping with stress strategies in the T-maze, heterozygous and NTg showed convergence of profiles. Differences in the latency to reach the intersection of the T-maze were found in spontaneous alternation test (*p* < 0.048, Bonferroni’s post hoc test, vs. heterozygous and NTg mice) and any of the homozygous mice accomplished the test completion criteria (*p* = 0.037, post hoc test vs. NTg). As detailed on the right, one NTg mice rested with their backs protected in the starting point, and one spent time but did not cross the intersection, the two others (75% of the sample) completed the test successfully. Among heterozygous, one of the animals turned but was not able to cross the intersection, the rest, 80% of the sample completed with the test completion criteria. Finally, 100% of homozygous mice, started but did not cross the intersection. In the working memory paradigm studied 24 h later all the homozygous animals failed to reach the acquisition criteria during the 5 min of the test. The test was prolonged to 10 min, but still, animals were unsuccessful (data not shown).

In the Morris water maze, cognitive deficits were the most salient distinctive trait in 3xTg-AD mouse survivors. As represented in Figure 6, cognitive deficits in spatial reference learning and memory differentiated the 19-month-old 3xTg-AD mice from age-matched NTg counterparts. In the assessment of visual perceptual learning and memory (Figure 6A), the three groups of mice showed the same mean escape latency on the first day of the cue task. On the second day, the platform was hidden and located in a reversed position; this made the new paradigm a difficult place task. Spatial reference memory assessed by means of place-learning showed differences between genotypes on the third day of the test (*p* = 0.002, Student’s *t*-test). Swimming velocities were not different between the groups (data not shown). 2 h 30 min after the last day of place learning, an extra trial with the removal of the platform indicated the worse performance of 3xTg-AD groups compared with NTg (Figure 6C). NTg mice invested more time than the other groups in the target quadrant (*p* = 0.046, Student’s *t*-test). Indeed, heat plots reveal that the NTg group mostly searched close to the designated platform position. Moreover, the latency of reaching the previous location was also recorded. In this case, the NTg mice reached the trained location faster than 3xTg-AD mice (*p* = 0.047, Student’s *t*-test). However, when the time the animals stood immobile during the removal test, considered as floating time, was recorded, the NTg and heterozygous groups showed a high prevalence of floating (50% (4/8) and 40% (2/5) respectively). In contrast, 3xTgAD mice maintained movement all the time during the test (0/4).

### 3.6. Peripheral Organs Pathological Status

#### 3.6.1. Splenic, Renal, and Hepatic Histopathological Evaluation

Figure 7 illustrates the results of the histopathological analysis of the spleen, kidney, and liver. Systemic amyloidosis damage was observed in the animals that presented lesions. Figure 7A shows representative images of microscopic analyses through Hematoxyline and Eosin staining. As detailed in Figure 7B, the incidence of amyloidosis was higher in the spleen (77%), kidney (100%), and liver (100%) 3xTg-AD mice than in NTg mice (38%, 38%, 25%, respectively).

In the spleen, amyloid deposit distribution was generalized. It was first located in the marginal zone of white pulp and progressively extended to all red pulp. This fact implies an intense hypocellularity and a functional loss of the organ. Although the presence of this damage was higher in the 3xTg-AD group, no significant statistical difference was achieved when we evaluated the injury score. As shown in Figure 2D, the spleen’s size and relative size (% vs. bodyweight) were recorded as an indirect measure of its physiological status. However, no differences between groups were observed (*p* > 0.05, Student’s *t*-test). Moreover, weight measures did not correlate with the severity of amyloidosis damage. 

In the kidney, the presence of amyloid was observed mainly in the glomerulus and occasionally in the tubular interstitium. In general, the score of the lesion was from moderate to very severe. Concurrently in the more severe cases, multifocal-generalized amyloid deposition was observed in the renal interstitium. Moreover, proteinuria was also observed as distended tubs with the presence of an intense eosinophilic colloid –protein. This damage could cause an important renal dysfunction. Genotype differences were observed when we evaluated the injury score (*p* = 0.046, U Mann Whitney). 

In the liver, the lesions affected centrilobular veins, portal vessels, or even higher range interstitial vascularization. Unlike what happened in the spleen and kidneys, the characteristics of the hepatic lesions were mild to moderate, with little pathological relevance. However, the results confirm the systemic nature of the disease since genotype differences were observed when we evaluated the injury score (*p* = 0.027, U Mann Whitney). 

As commented, this amyloid damage did not affect all organs equally and presented some heterogeneity. The spleen and kidney presented substantial damage, whereas were mild and little relevant in the liver. However, a correlation between the three organ lesions’ intensities was observed (*p* < 0.001, Spearman’s correlation). Furthermore, as detailed in Figure 7C, genotype differences were observed when we evaluated the general systemic total score (*p* = 0.019, Student’s *t*-test).

#### 3.6.2. Oxidative Stress Parameters in Peripheral Organs

Concerning the antioxidant capacity from the homogenization of different organs (spleen, kidneys, liver, and heart) no statistical differences were observed in the levels of total glutathione (Figure 8A), as well as the enzymatic activity of glutathione peroxidase (Figure 8B) and glutathione reductase (Figure 8C).

## 4. Discussion

The present study was aimed to study whether the AD-genotype load can modulate the pathological burden, behavioral phenotype, and neuro-immunoendocrine status in a singular cohort of long-lived (19-month-old survivors) heterozygous and homozygous male 3xTg-AD and as compared to age-matched non-transgenic controls. Therefore, a comprehensive screening of several physical, emotional, and cognitive functions was successively performed using a battery of tests. First, we determined their physical status, including frailty and sensorimotor function, and the survival of animals being continuously monitored from birth to the end of the experiment. Second, the brain was evaluated by levels of amyloid precursor protein (APP), synaptophysin as a synaptogenesis and neuroplasticity marker, and choline acetyltransferase, the enzyme responsible for the Ach synthesis. Third, the behavioral phenotype of animals was evaluated to determine their emotional and anxiety-like profiles, and cognitive functions were assessed in spatial learning and memory tasks. Fourth, neuro-immunoendocrine crosstalk was characterized by glucocorticoid levels, an indicator of HPA axis function. Finally, the histopathological evaluation, and oxidative stress parameters of the spleen, kidney, liver, and heart were used to assess the systemic health of peripheral organs.

### 4.1. Convergence of Physical Status and Survival Profiles of Long-Lived Survivors

Exclusion or under-representation of older individuals is not unusual in clinical trials despite being the most significant health care resources [43]. Concretely, in clinical trials on Alzheimer’s disease, the patients enrolled are not representative of their general population [44]. This problem also occurs at the preclinical level, where most experimental designs are performed in young adulthood, adults, and middle-aged animals. Heterogeneity found in the complexity of age-related scenario and the reduced survival of animals, with the concomitant increase of laboratory costs, produce scarcity research in very old mice, even more in models of neurodegenerative disease [19,45,46]. 

In the 3xTg-AD mice model, a frailty/survival paradoxical was described, with female exhibiting a worse neuropathological status [47] but higher mortality rates in homozygous [26,28] and heterozygous male 3xTg-AD mice [48]. However, the present cohorts present an extraordinary survival rate, higher than 70% at 19 months of age. In this singular scenario, we were interested to study the relevance of AD-genotype load and the frailty/survival paradigm in normal and neurodegenerative pathological aging.

Frailty, a common tool to measure health status, is becoming widely used in clinical decision making as disease outcomes such as Covid-19 or even mortality were better predicted by frailty index than age or comorbidity [9,49]. In mouse models, the Mouse Clinical Frailty Index [37], a translational adaptation of frailty index data in humans, is also a valuable tool in longevity and aging studies in mice. Although we have noticed and increased frailty levels in 14-month-old 3xTg-AD males compared to NTg counterparts [50], no significant differences were observed in this cohort of 19-month-old survivors. The higher scores were primarily observed in the integument and muscular-skeletal dimension, in accordance with the more common clinical presentations in aged mice, such as alopecia and dermatitis [51]. 

The body weight of animals was also monitored as an index of health/frailty status, and, in this case, the overweight characteristic of the 3xTg-AD Spanish colony was not found [52]. Instead of this, reduced body weight was observed predominantly in homozygous mice, similarly as described previously in isolated 3xTg-AD male mice [53].

Regarding sensorimotor function, and as observed in 18-month-old transgenic and NTg females [19], poor physical motor abilities were observed in both groups. A convergence of sensorimotor profiles was caused by lack of coordination and short latencies of falling with high individual variability.

### 4.2. Non-Linear HPA-Axis Activation in 3xTg-AD Males

A crosstalk between endocrine abnormalities of the hypothalamic-pituitary-adrenal (HPA) system and patients with Alzheimer’s disease have been described repeatedly [54]. Elevated cortisol levels have been associated with cognitive decline dementia [55] and peripheral immunodepression [56]. These data agree with our first report in 15-month-old animals, where a significant increase in plasma corticosterone was observed in 3xTg-AD male mice, suggesting enhanced HPA axis activation accompanied with immune function alterations [28]. In the present study, we described a non-linear increase of corticosterone levels, with heterozygous mice presenting higher levels than NTg mice. This can be explained as cortisol levels seem to be associated with the progression of the disease rather than the severity. Thus, Csernansky et al. observed more significant correlations in AD-patients at the early stages of the disease [57]. It has been also described the role of microglia in the chronic-stress induced neuroinflammation and their contribution to neurodegenerative disease [58,59]. Study the impact this HPA axis hyperactivation on inflammation and further activation of glial cells would be necessary to better understand the underlying mechanism of the cognitive and anxiety-like symptoms observed in 3xTg-AD, opening new preventive and prognostic options. 

### 4.3. Amyloid Precursor Protein (APP) Levels Increased in a Genetic-Load-Dependent Manner but Age-Dependent Convergence of Synaptophysin and Choline Acetyltransferase Brain Levels

Alzheimer’s disease is defined as an accumulation of amyloid-β (Aβ) plaques and tau-containing neurofibrillary tangles (NFTs), although they can also be found in normal aging. Neuroinflammation and other metabolic and neuronal processes such as synaptic changes and changes in neurotransmitter systems also play a role in the pathogenesis of AD [2]. The 3xTg-AD model develops age-related, progressive neuropathology, including plaques and tangles mimicking human patients’ temporal and neuroanatomical patterns [20,21]. In the current experimental scenario with survivors, we can study how the three different levels of AD-genetic-load (null, heterozygous, homozygous) translate into the expression of these hallmarks of AD. 

The hippocampus was the most sensitive AD-target region to show the effect of genetic-load in APP levels, while in the cortical areas studied (prefrontal and entorhinal), the difference did not reach statistical significance, probably due to the variability. These results agree and complement those reported in young mutants in the original work describing the model [21].

Synaptophysin is a membrane protein of synaptic vesicles closely related to cognitive processes and synaptic plasticity [60]. Several authors have reported changes in synaptophysin expression in Alzheimer’s patient’s brain areas [61,62]. In 3xTg-AD mice, we have already reported that synaptophysin expression levels significantly decreased in middle-aged mice [22,63]. However, no significant genotype differences were observed in survivors studied in the present report.

Similar results were observed when we evaluated the levels of ChAT, the enzyme responsible for acetylcholine synthesis, one of the most involved neurotransmitters in the disease. Nowadays, acetylcholinesterase inhibitors still constitute the most important group of drugs for Alzheimer’s disease treatment [64]. Despite the significative decreased levels observed in middle-aged mice [27,65], no significant differences were observed in these group of 19-month old survivors. Interestingly, aging-related loss of presynaptic protein synaptophysin and cholinergic inputs observed in C57BL/6J male mice [66] could explain that aging processes might be related to this convergence of synaptophysin and ChAT levels. 

Therefore, amyloid levels were increased in a genetic-load-dependent manner independently of aging. However, synaptic, and cholinergic functions seem to be more dependent on aging processes/survival paradigms. 

### 4.4. Genotype Load Modulates Anxiety-Like Patterns Despite Similar Cognitive Impairments

It has been reported that genetic load can aggravate the extent and accelerate the onset of pathological alterations in transgenic mice [67,68]. For example, it has been previously established age-dependent difference in pathology and cognitive deterioration between hemizygous and homozygous 3xTg-AD mice [21,30,69]. The present work aimed to explore the genetic-load-dependent effect on behavioral and psychological outcomes in a singular cohort of long-lived mice. Studying the impact of the genetic component at advanced stages of the disease may help to understand better the aging interactions which can be involved in the wide heterogeneity and complexity of patients’ clinical profiles.

As previously observed in 18-month-old female survivors [19], no genotype differences were observed between 3xTg-AD and NTg in the corner test, open-field test, and T-maze, three classical unconditioned tests measuring neophobia, exploratory activity, and emotionality [18] neither in the dark and light box and marble test. This convergence of behavioral profiles involves considering the contribution of genetic patterns and/or aging-related decline per *se*. In this sense, our laboratory has described convergence of profiles in the context of poor aging from 12 to 18 months of age as part of the complexity of age-related scenarios and heterogeneity among all populations, including both wild-type mice and 3xTg-AD mice [19,50,53]. The survival of very old 3xTg-AD mice points to the existence of distinct brain and systemic physiological protective mechanisms for AD-pathology besides those that may exist in normal aging [50,70]. 

However, on the other hand, when we evaluated independently homozygous and heterozygous mice, we observed that genotype load modulated anxiety-like- profiles. When we repeated the open field test 24 h later, the ethogram exhibited higher latencies. Thus, increases were observed in the latency to exit the center, entrance to the periphery and vertical activity in homozygous, and spent more time in the center than heterozygous mice. In agreement with previous reports showing a 24 h long-term memory deficit in 3xTg-AD mice at 2, 4, 6, and 14 months of age, the behavioral response of mutants did not benefit from previous experience in contrast to NTg age-matched counterparts [35,50]. However, in this case, repeated the test increased the genetic-load differences not observed in the first day-test.

In the dark and light box test, the anxiogenic profile of homozygous 3xTg-mice was associated by a delay in the risk assessment activity and the number of crossings and rearings performed in the dark area. 

The anxiogenic profile was also confirmed in the T-maze test, indicating that the profile that have been previously described in this model [18,29,31] persists in 19-month-old survivors. Noteworthy, none of the homozygous mice accomplished the test completion nor acquisition criteria. This fact indicates their aged status and/or poor motivation [19], a singular fact that we have also observed associated with severe status neurodegenerative models [71]. Moreover, the increased latencies of homozygous to achieve the crossing intersection have been related to immunosenescence and reduced survival [72]. 

As reported in 18-month-old female survivors [19], cognitive impairment remained the salient distinctive trait in 3xTg-AD mice as compared to NTg mice. These results are in accordance with worse performance in Morris Water Maze observed in several ages of 3xTg-AD [18,29,31,35,73]. On the other hand, the swimming performance can reflect their emotional status in an aquatic environment known to be anxiogenic for mice [74]. In this case, the floating behavior (inactivity without forward movement) characteristic of non-transgenic performance [75] was not presented in 3xTg-AD homozygous mice. However, it was observed in non-transgenic and 3xTg-AD heterozygous mice. Therefore, these results allow us to propose that the underlying mechanisms are still preserved in heterozygosis. The results in genetic load also confirmed the anxiogenic and cognitive phenotypes being modulated independently in both groups. 

These results highlight the distinct contribution of genetic-load disease-related components and aging to behavioral readouts. Cognitive impairment is a distinct trait of the disease. However, anxiety-like behavior seems to be more related to genetic-load, been more affected the animals with higher genetic dosages. Despite the inherent limitations of the sample size of the present study, consistent behavioral patterns make the present results of interest for further exploration in a larger size sample. These data agree with the premorbid predisposition to anxiety and depression in familial Alzheimer’s disease [76] and may explain why early-onset Alzheimer’s disease patients more often present non-amnestic phenotypic variants [77].

### 4.5. Peripheral Organs Presented Histopathological Alterations but No Differences in Their Oxidative Stress Parameters

Neurodegenerative disorders such as dementia are associated with increased mortality compared to the general old population [7,8]. Clinical evidence suggests an interaction between the brain and systemic abnormalities that can explain this fact [78]. For instance, it has been repeatedly described that the immune system has an important role in AD pathology, both at the central nervous system and peripheral level [79,80]. Our research in the 3xTg-AD mice also supports the relevance of the neuro-immunoendocrine impairment in AD. Thus, we have described significant involvement of the peripheral immune system [28,81,82,83,84,85]. The impairment was also monitored through peritoneal cells in a longitudinal study from 2 to 15 months of age, mimicking premorbid, prodromal, early to advanced stages of the disease [84]. Therefore, a better understanding of neuro-immunoendocrine crosstalk could help with an early diagnosis and, as we have proposed, improve the disease monitoring of AD.

In the present study, histological analysis was performed on the spleen, kidney, and liver. Systemic amyloidosis damage was observed in the animals that presented lesions. A higher incidence was observed in 3xTg-AD mice independently of genetic-load than in NTg mice. In the spleen, amyloid deposit distribution was generalized, producing an intense hypocellularity and a functional loss of the organ. In the kidney, the presence of amyloid was observed mainly in the glomerulus, causing an important renal disfunction. Although in the liver, the lesions presented little pathological relevance, it confirmed the systemic nature of amyloidosis. Amyloid has been reported to occur spontaneously in a variety of animal species, including mice. For instance, glomerular amyloidosis is common in older mice [51]. Moreover, the grade and incidence of amyloid deposition seem to increase with age. In SAM mice, a mouse model of senescence-accelerated mouse, renal amyloidosis was more frequent in animals with complications such as abscess, skin ulcer or tumors [85]. The easy measurement of their weight and relative weight (organometrics), with clinical translation, can be used as early indicators of peripheral immunological system aging [82,83,86], as also confirmed by other laboratories [87,88]. In this case, spleen weight was recorded as an indirect measure of their physiological status. However, no differences between groups were observed. Moreover, this measure did not correlate with the severity of amyloidosis damage. Similarly, in the SAM mice, the kidney/body weight ratio did not parallel the grade of renal amyloidosis [85].

Although brain oxidative stress in AD is accepted, the contribution of the disease to peripheral oxidative redox state has been scarcely studied. We have previously shown sex-specific immuno-endocrine aging in 3xTg-AD mice. Concretely, peripheral alterations in early oxidative stress status in male and female 3xTg-AD mice, with a decrease in antioxidant defenses and an increase in xanthine oxidase activity in most peripheral tissues, among them the spleen, kidney, and liver [28,52,86,89]. However, like the present study, no genotype differences were found in reduced glutathione levels in peritoneal leukocytes at 15 months of age [84]. These studies suggest a premature immunosenescence in the prodromal stage of AD. However, a decrease in antioxidants and an increase in oxidants associated with the aging process [90] could explain this convergence of profiles in our survivors. 

Taking all these data into account, the present study provides an interesting translational scenario showing complex neuro-immunoendocrine crosstalk. Furthermore, vulnerability/compensatory mechanisms in transgenic mice were observed as histopathological alterations showed organs dysfunction with no correlations in frailty index nor oxidative stress parameters.

## 5. Conclusions

The singular cohort of long-lived (19-month-old survivors) heterozygous and homozygous male 3xTg-AD mice studied here indicates that the AD-genotype load modulates the brain and peripheral pathological burden, behavioral phenotypes, and neuro-immunoendocrine status, compared to age-matched non-transgenic controls. The main findings pointed at the non-linear impact of genetic load in the different dimensions studied. While amyloid precursor protein (APP) levels increased in a genetic-load-dependent manner, synaptophysin and choline acetyltransferase brain levels referring to synaptic function were similar in the three groups of mice, that may agree with the decrease of synaptic function described in aged animals. Cognitive impairment and the level of activation of the HPA-axis were salient traits in both 3xTg-AD survivor groups, with no impact of genetic load. In contrast, homozygous and heterozygous exhibited different responses in classical unconditioned anxiety tests. Homozygous 3xTg-AD mice showed severe hypofunction in most tests, while heterozygous resembled controls in some anxiety variables and risk assessment, suggesting different genetic-load modulation of these states. Complex neuro-immunoendocrine crosstalk was also observed. Bodyweight loss and splenic, renal, and hepatic histopathological injury scores provided evidence of the systemic features of AD, despite similar peripheral organs’ oxidative stress. The present study provides an interesting translational scenario to study further genetic-load and age-dependent vulnerability/compensatory mechanisms in Alzheimer’s disease. Research with very old mice and, particularly, in long-term survivors’ cohorts with specific behavioral and physiological profiles can be helpful for the better understanding of heterogenous manifestations reported in end-of-life Alzheimer’s patients. 

## Figures and Tables

**Table 1:** Similarity of physical status and sensorimotor function in 19-month-old male 3xTg-AD mice compared to sex- and age-matched NTg mice with normal aging. Results are expressed as mean ± SEM or incidence. Student’s *t*-test, n.s., no statistically significant; *p* > 0.05.

	Males, 19-Month-Old			
	NTg Mice *n* = 8	3xTg-AD Mice *n* = 9	Genotype Differences	Genetic Load Differences
**Physical Status, Reflexes, and Sensorimotor Function**				
*Frailty index score*	0.05 ± 0.02	0.03 ± 0.01	n.s.	n.s.
*Reflexes*				
*Visual placing reflex (3 trials)*	3/3	3/3	equal	equal
*Posterior leg reflex (3 trials)*	3/3	3/3	equal	equal
*Wood rod test (two 20-s trials)*				
Equilibrium (mean falling latency, s)	3.63 ± 1.18	1.94 ± 1.01	n.s.	n.s.
Coordination (mean distance, cm)	0.19 ± 0.09	0.06 ± 0.05	n.s.	n.s.
*Wire rod test (two 20-s trials)*				
Equilibrium (mean falling latency, s)	6.19 ± 2.32	7.78 ± 2.40	n.s.	n.s.
Coordination (mean distance, cm)	0.19 ± 0.19	0.28 ± 0.12	n.s.	n.s.
*Wire hang test (two 5-s trials)*				
Strength (mean time hold, s)	2.50 ± 0.33	3.50 ± 0.53	n.s.	n.s.
Coordination (mean distance, segments)	0 ± 0	0 ± 0	equal	equal
*Wire hang test (one 60-s trial)*				
Resistance (time hold, s)	29.5 ± 8.57	29.11 ± 9.33	n.s.	n.s.
Coordination (distance, segments)	1.63 ± 0.56	0.89 ± 0.39	n.s.	n.s.

**Table 2:** Similarity, exploratory and BPSD-like domains, in 19-month-old male 3xTg-AD mice as compared to sex- and age-matched NTg mice with normal aging. Results are expressed as mean ± SEM. Student’s *t*-test, ** *p* < 0.01, vs. NTg mice; n.s., no statistically significant, *p* > 0.05.

	Males, 19-Month-Old			
	NTg Mice *n* = 8	3xTg-AD Mice *n* = 9	Genotype Differences	Genetic Load Differences
**BPSD-Like Behaviors and Exploratory Activity**				
*Corner test (one 30-s trial)*				
Vertical activity (latency, s)	19.13 ± 3.78	14.89 ± 3.02	n.s.	n.s.
Vertical activity (number)	1.25 ± 0.41	2.22 ± 0.62	n.s.	n.s.
Horizontal activity (number)	6.13 ± 0.48	3.44 ± 0.50	**	n.s.
*24 h Corner test (one 30-s trial)*				
Vertical activity (latency, s)	25.50 ± 2.97	19.89 ± 3.65	n.s.	n.s.
Vertical activity (number)	0.75 ± 0.53	1.00 ± 0.33	n.s.	n.s.
Horizontal activity (number)	2.88 ± 0.51	3.22 ± 0.49	n.s.	n.s.
*Open field test (5 min)*				
Initial freezing (latency, s)	5.25 ± 1.25	5.33 ± 0.69	n.s.	n.s.
Exit of the center (latency, s)	11.13 ± 1.80	11.56 ± 2.06	n.s.	n.s.
Entrance to the periphery (latency, s)	100 ± 43.00	82.22 ± 41.49	n.s.	n.s.
Vertical activity (latency, s)	199 ± 41.88	173.56 ± 40.66	n.s.	n.s.
Self-grooming (latency, s)	190.1 ± 28.89	147.44 ± 23.45	n.s.	n.s.
Vertical activity (number)	See Figure 4A			
Horizontal activity (distance, m)	See Figure 4A			
Time immobile (s)	194.35 ± 27.91	224.89 ± 17.00	n.s.	n.s.
Time in periphery (s)	141.42 ± 38.53	151.02 ± 37.05	n.s.	n.s.
Self-grooming (number)	1.13 ± 0.35	1.11 ± 0.20	n.s.	n.s.
Defecation boli (number)	3.875 ± 0.69	2.67 ± 0.62	n.s.	n.s.
*24 h Open field test (5 min)*				
Initial freezing (latency, s)	See Figure 4B			
Exit of the center (latency, s)	See Figure 4B			
Entrance into the periphery (latency, s)	See Figure 4B			
Vertical activity (latency, s)	See Figure 4B			
Self-grooming (latency, s)	See Figure 4B			
Vertical activity (number)	See Figure 4A			
Horizontal activity (distance, m)	See Figure 4A			
Time immobile (s)	234.77 ± 18.76	235.66 ± 19.89	n.s.	n.s.
Time in periphery (s)	See Figure 4C			
Self-grooming (number)	1.00 ± 0.189	0.89 ± 0.261	n.s.	n.s.
Defecation boli (number)	3.88 ± 0.479	2.89 ± 0.655	n.s.	n.s.
*Marble test (30 min)*				
Intact marbles (number)	2.50 ± 1.23	3.67 ± 1.11	n.s.	n.s.
Rotated marbles (number)	2.88 ± 0.63	2.89 ± 0.67	n.s.	n.s.
Half-buried marbles (number)	2.13 ± 0.71	1.67 ± 0.50	n.s.	n.s.
Buried marbles (number)	1.50 ± 0.53	0.78 ± 0.50	n.s.	n.s.
